# Micro RNAs as a Diagnostic Marker between Glioma and Primary CNS Lymphoma: A Systematic Review

**DOI:** 10.3390/cancers15143628

**Published:** 2023-07-14

**Authors:** Mohammad Amin Dabbagh Ohadi, Mir Sajjad Aleyasin, Reza Samiee, Sanaz Bordbar, Seyed Farzad Maroufi, Nikoo Bayan, Sara Hanaei, Timothy R. Smith

**Affiliations:** 1 Department of Pediatric Neurological Surgery, Children’s Medical Center, Tehran University of Medical Sciences, Tehran 1419733151, Iran; ma-dohadi@student.tums.ac.ir (M.A.D.O.); farzadmaro@gmail.com (S.F.M.); 2 Interdisciplinary Neuroscience Research Program, Tehran University of Medical Sciences, Tehran 1417755331, Iran; 3 Students’ Scientific Research Center, Tehran University of Medical Sciences, Tehran 1417755331, Iran; ms.aleyasin2020@gmail.com (M.S.A.); rezasamieepgh@gmail.com (R.S.); s-bordbar@student.tums.ac.ir (S.B.); nikoo.bayan1999@gmail.com (N.B.); 4 Neurosurgery Department, Imam Khomeini Hospital Complex (IKHC), Tehran University of Medical Sciences, Tehran 1419733151, Iran; sara.hanaei@gmail.com; 5 Department of Neurosurgery, Brigham and Women’s Hospital, Boston, MA 02115, USA

**Keywords:** glioma, primary central nervous system lymphoma, liquid biopsy, microRNA, biomarkers, diagnosis, discrimination, miR-21, miR-16, miR-205, miR-15b, miR-301, miR-711, glioblastoma

## Abstract

**Simple Summary:**

Differentiating between glioma and primary central nervous system lymphoma (PCNSL) is difficult, and current diagnostic methods are of limited efficacy. Liquid biopsies that detect circulating biomarkers, such as microRNAs (miRs), may provide valuable insights into diagnostic biomarkers. In this review, a systematic search was conducted, and 16 dysregulated miRs were identified, including miR-21, which was the most prominent miR with higher levels in PCNSL, followed by the glioma and control groups. The lowest levels of miR-16 and miR-205 were observed in glioma, followed by PCNSL and control groups, whereas miR-15b and miR-301 were higher in both tumor groups, with the highest levels observed in glioma patients. The levels of miR-711 were higher in glioma (including GBM patients) and downregulated in PCNSL compared to the control group. Using these six circulating microRNAs as liquid biomarkers with unique changing patterns could aid in better discrimination between glioma and PCNSL.

**Abstract:**

Differentiating glioma from primary central nervous system lymphoma (PCNSL) can be challenging, and current diagnostic measures such as MRI and biopsy are of limited efficacy. Liquid biopsies, which detect circulating biomarkers such as microRNAs (miRs), may provide valuable insights into diagnostic biomarkers for improved discrimination. This review aimed to investigate the role of specific miRs in diagnosing and differentiating glioma from PCNSL. A systematic search was conducted of PubMed, Scopus, Web of Science, and Embase for articles on liquid biopsies as a diagnostic method for glioma and PCNSL. Sixteen dysregulated miRs were identified with significantly different levels in glioma and PCNSL, including miR-21, which was the most prominent miR with higher levels in PCNSL, followed by glioma, including glioblastoma (GBM), and control groups. The lowest levels of miR-16 and miR-205 were observed in glioma, followed by PCNSL and control groups, whereas miR-15b and miR-301 were higher in both tumor groups, with the highest levels observed in glioma patients. The levels of miR-711 were higher in glioma (including GBM) and downregulated in PCNSL compared to the control group. This review suggests that using these six circulating microRNAs as liquid biomarkers with unique changing patterns could aid in better discrimination between glioma, especially GBM, and PCNSL.

## 1. Introduction

The most common malignant primary brain tumor in adults is considered to be glioma [1]. The conventional diagnostic methods for detecting glioma rely on neurological examination, neuroimaging, and pathological studies [2]. Gliomas make up 80% of brain tumors, with an incidence of 6 cases per 100,000 people per year in the United States [3]. Surgery is the primary treatment for gliomas and the five-year survival rate for low-grade gliomas ranges from 50% to 90%, while high-grade gliomas, especially glioblastoma (GBM), have a survival rate of less than 10% [3,4]. Primary central nervous system lymphoma (PCNSL) is a non-Hodgkin lymphoma that constitutes about 2% of primary brain tumors [5]. Treatment for PCNSL primarily involves nonsurgical approaches, including a combination of chemotherapy, radiation therapy, and targeted therapy [5]. 

Differentiating higher grades of glioma from PCNSL has been considered an issue for neurosurgeons, as the symptoms and imaging features of both have many similarities. However, accurate differentiation is crucial as the treatment approaches for these two conditions are vastly different. Furthermore, tissue biopsy can be nondiagnostic in 10% of PCNSLs. MRI and dynamic susceptibility-weighted contrast-enhanced MRI can be regarded as diagnostic methods to differentiate between these two tumors, although not always [6,7]. With the advance of diagnostic measures, biomarkers are becoming a diagnostic modality for improved brain tumor differentiation. Several studies have shown that screening for certain RNA strands can have a high specificity in the diagnosis of GBMs [8]. Meanwhile, several prior studies have indicated that CSF RNAs have the potential to act as diagnostic biomarkers for PCNSL [9,10].

MicroRNAs (miRs) are small non-coding RNAs (ncRNAs) [11] involved in many biological processes such as cell growth, cell development, and the regulation of many cellular functions [12]. Many studies have shown miR expression to be involved in various pathogenesis events in brain tumors [13]. The levels of these non-coding RNAs are associated with prognosis and therapeutic outcomes in brain tumors [14]. The microRNA miR-21, one of the most studied miRs, is linked to radio- and chemoresistance in glioblastoma [14]. Inhibitors of miR-21 have shown promise in increasing chemosensitivity, particularly when combined with temozolomide, by suppressing growth and promoting apoptosis [15]. In addition, evaluating the expression level of miRs in patients could be a strategy for the early detection of multiple diseases, including neoplastic processes. MicroRNAs could be available in the tissues, cells, CSF, and blood. Hence, the retrieval of samples for microRNA analysis could be minimally invasive compared to biopsies [16,17]. Here, we aim to systematically review the current literature on the diagnostic value of miRs in differentiating PCNSL from glioma and GBM.

## 2. Method

### 2.1. Search Strategy and Selection Criteria

On 14 March 2022, a systematic search was conducted. Additionally, an updated search was performed on 8 February 2023, using the PRISMA guidelines [18] to identify articles that evaluated the expression levels of miRs in both GBM and PCNSL patients. The Scopus, Web of Science (WOS), Embase, and PubMed databases were searched using the search strategies provided in Supplementary Table S1. Two independent reviewers (R.S. and M.A.) screened the articles, and any disagreements were referred to the third reviewer (S.B.). Finally, citations of included articles were reviewed to find any non-included related articles. The inclusion criteria were as follows: (1) published original studies available in English (excluding case reports), (2) sampling of blood or CSF, and (3) providing information on the level of miRs in PCNSL and glioma or GBM patients. The exclusion criteria were as follows: (1) reviews and meta-analyses, (2) animal studies, (3) studies with unclear data on the patients and the miRs employed, and (4) studies that used invasive sampling methods such as biopsy.

### 2.2. Data Extraction

The following data were extracted: patients’ age and gender, information regarding the methodology and sample collection, and any data on expression levels, such as AUC, sensitivities, specificities, and cut-offs. Additionally, graphs and figures were utilized that depicted the miR change patterns including those that distinguished PCNSL from GBM or gliomas. The extraction process was conducted by two authors (R.S. and M.A.).

### 2.3. Enrichment Analysis

In this study, to explore the possible pathways and functions of the miRs, the StarBase v2.0 online platform (http://starbase.sysu.edu.cn/, accessed on 7 January 2023) was used to identify the target genes of the miRs of interest. A KEGG pathway analysis using the target genes to identify the enriched pathways associated with miRs showed significant level changes and included a control group in their related study. To visualize the results of the KEGG analysis, enrichment plots were generated using the SRplot online platform (http://bioinfo.uth.edu/SRPlot/, accessed on 7 January 2023). The enrichment plots allowed us to easily identify the significantly enriched pathways associated with the target genes of the miRs.

## 3. Results

**Figure 1:**
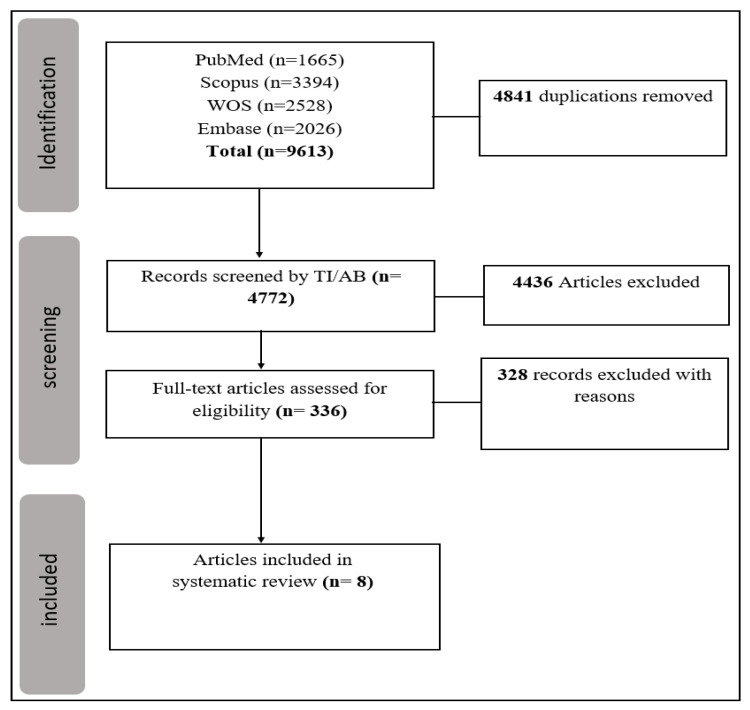
Prisma flowchart.

The designated search string resulted in 9613 articles. After the removal of duplicates, 4772 records were screened for title and abstract. Full-text screening of 336 articles excluded 328 papers (13 review articles, 38 articles with unclear data, 72 animal studies, 107 articles with irrelevant data, and 98 in vitro studies). Finally, 8 articles remained for the quantitative synthesis. The Prisma flow chart is presented in Figure 1. Sixteen dysregulated miRs were found with significant level changes in glioma and PCNSL. All studies employed qRT-PCR for miR measurement. Additionally, all specimens were collected from plasma/serum, except for two samples which were collected from cerebrospinal fluid (CSF) (Table 1). 

### 3.1. Serum

Six studies used serum samples. Among these, miR-21 was the most studied miR which was found to be higher in both the PCNSL and GBM groups compared to controls (*p*-value < 0.0001, area under curve (AUC) > 85%) [19]. Yang et al. found higher levels of miR-21 in PCNSL compared to GBM and both were higher than controls [20]. Another study by Si et al. showed 10 microRNAs (miR-6820-3p, miR-6803-3p, miR-4756-5p, miR-30a-3p, miR-548am-3p, miR-487a-3p, miR-3918, miR-4751, miR-371a-3p, and miR-146a-3p) to be significantly (*p*-value < 0.05) elevated in PCNSL compared to GBM without reaching an AUC of over 80% [23]. The miR-301 level in glioma was higher than PCNSL, and both were higher compared to healthy controls (*p*-value < 0.01) [24]. The miR-205 level in glioma was less than PCNSL and both were less than in healthy controls (*p*-value < 0.01) [25]. In the study, it was observed that the levels of miR-16 were notably lower in glioma as compared to various neurological disorders (VND) included in the research (*p*-value = 0.02). In another study, mir-15b and mir-21 levels were higher in glioma than VND (*p*-value < 0.01), and a detailed level-change comparison of these miRs in three groups was performed, as mentioned in Table 1 [22].

### 3.2. CSF

Two studies investigated CSF samples. The microRNA miR-15b was higher in glioma compared to PCNSL and both were higher than controls, which also correlates with its amount in plasma (*p*-value < 0.05) [27]. A study by Drusco et al. explored five microRNAs: miR-451, miR-125b, and miR-223 were higher in glioma compared to PCNSL and controls (microRNA concentration = 11.75, 9.72, 13.24). The miR-711 level in PCNSL was lower than controls, and GBM (microRNA concentration = 9.54). The miR-935 level decreased in PCNSL, while it did not express in GBM [21].

## 4. Discussion

**Figure 2:**
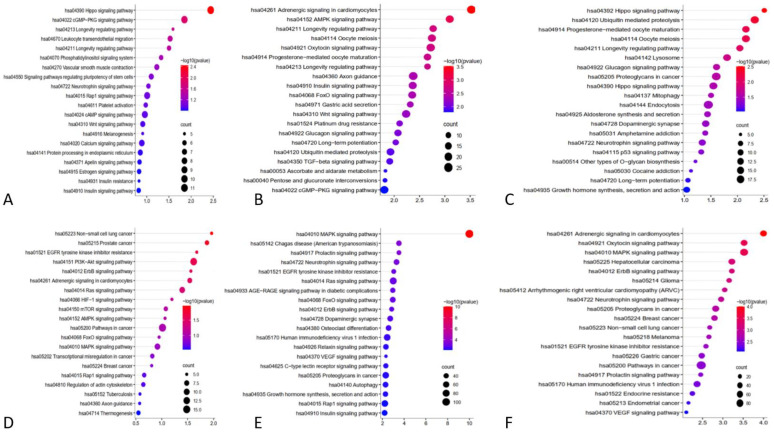
KEGG pathway analysis using the targeting genes to identify the enriched pathways associated interested miRs. (**A**): miR-21, (**B**): miR-15b, (**C**): miR-16, (**D**): miR-301, (**E**): miR-711, (**F**): miR-205.

In this study, a systematic review of the literature was carried out to identify miRs that have sufficient evidence of their role in differentiating between glioma and PCNSL. In total, 20 miRs were identified with distinct expression patterns in glioma (including GBM) and PCNSL. Among the miRs of interest, 13 were found to be higher in PCNSL, while 3 were higher in glioma, including GBM in one study. The control group demonstrated significant level changes in 6 out of the 16 microRNAs, suggesting their potential role in discriminating between these two tumors. Here, we provide an overview of these 6 miRs, their roles in tumorigenesis, and associated well known pathways found in KEGG analysis (Figure 2), as well as their potential for differentiating between glioma and PCNSL.

### 4.1. miR-21

The microRNA miR-21 is a well-known oncogenic miR that is over-expressed in various types of tumors [26,28]. Its upregulation has also been reported in the cerebrospinal fluid (CSF) of patients with primary central nervous system lymphoma (PCNSL) [9,26]. According to Baraniskin et al., miR-21, along with miR-19b and miR-92, can differentiate PCNSL from other neurological disorders (headache, epilepsy, syncope, and stroke) with a sensitivity of 97.4% [9]. Moreover, miR-21 is upregulated in GBM cases, suggesting its potential as a diagnostic biomarker [29,30].

**Figure 3:**
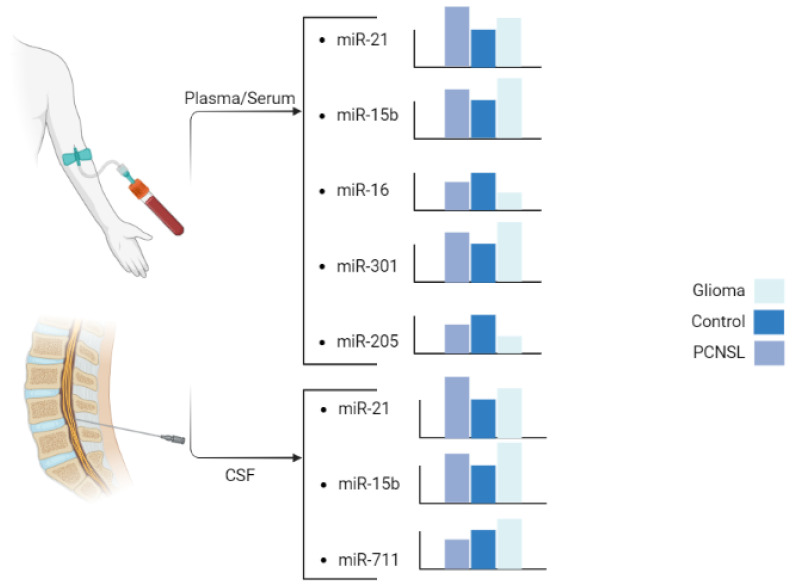
Six miRs of interest with significant and unique patterns of change. Created in BioRender.com (accessed on 15 May 2023).

In a study by Baraniskin et al., CSF levels of miR-21 were found to be higher in PCNSL cases than in GBM cases, and both were higher compared to the VND (non-neoplastic neurological disorders) group. They also reported that miR-15 and miR-21 have a sensitivity of 90% and a specificity of 100% for differentiating GBM from PCNSL cases [27]. Furthermore, the level of miR-21 in the serum of GBM and PCNSL patients compared to healthy controls was found to be significant, suggesting its potential as a serum biomarker for differentiating between PCNSL and GBM [19]. D’urso et al. also used blood to differentiate glioma from PCNSL and reported results consistent with the expression levels of miR-21 reported in other studies [22]. Later, Yang et al. confirmed these results in plasma with a larger sample size [20]. These studies suggest that comparing miR-21 expression levels in cerebrospinal fluid (CSF) and blood may enable differentiation between PCNSL and GBM, exhibiting a high discriminatory ability (AUC > 80%) with this specific pattern (Figure 3).

**Figure 4:**
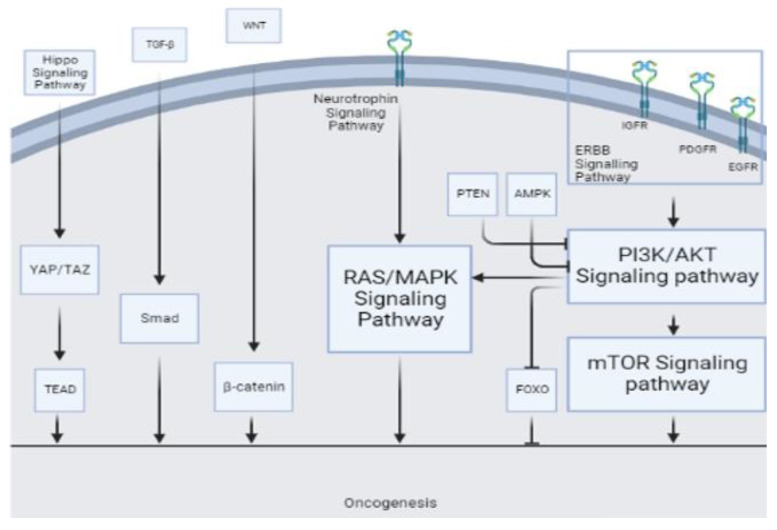
Various tumorigenesis pathways serve as sites of action for different miRs.

The microRNA miR-21 has been found to be upregulated in many normal tissues, where it participates in maintaining tissue homeostasis and normal cellular functions. It regulates the expression of target genes involved in various signaling pathways, such as those related to growth factor signaling, apoptosis, and inflammation. Additionally, miR-21 is also known to be dysregulated in many cancers; miR-21 prevents apoptosis and regulates cellular growth and differentiation [31,32,33,34,35]. It targets various genes and pathways, including the tumor suppressor gene P53 [36], transforming growth factor-β (TGF-β) which can regulate Smad7 [36,37], and mitochondrial apoptosis pathways [36]. Other apoptotic targets of miR-21 include the phosphate and tensin homologous (PTEN) protein [33,34,38,39,40], programmed cell death gene 4 (PDCD4) [32,38,41], and tropomyosin 1 [36,42]. Chai et al. reported that miR-21 regulates glioma proliferation and inhibits apoptosis through decreasing PTEN and increasing PI3K/AKT [43] (Figure 4). The increase in miR-21 expression may cause the inhibitors of matrix metalloproteinase (MMPs), such as RECK [38,44] and TIMP3 [34] to decrease, leading to elevated MMPs and an increased invasiveness of glioma tumor cells [45]. Li et al. suggest that miR-21 targets leucine-rich repeat interacting protein 1 (LRRFIP1), which inhibits NF-κB signaling [46]. NF-κB plays a crucial role in various cancer-related pathways, particularly in glioma and PCNSL, where it is prominently associated with tumor growth and invasion. This pivotal role has positioned NF-κB as a promising therapeutic target in these malignancies [47,48]. Moreover, certain studies have reported that the downregulation of miR-21 inhibits the epidermal growth factor receptor (EGFR) and AKT pathways [34] (Figure 4). Kwak et al. reported that miR-21 is involved in Spry2 reduction in high-grade human glioma tissue, leading to the disruption of the Ras/MAPK signaling negative feedback circuit mediated by Spry2 and responsible for glioma invasion [49] (Figure 4). Previous reports have shown that WNT1 represses miR-21, indicating its involvement in the WNT/β-catenin pathway [50,51]. This pathway is involved in the proliferation and migration of glioma and hematologic malignancies [52,53]. Ables et al. reported that inhibiting Btg2 via miR-21 may lead to increased microglia proliferation [40]. Btg2 is involved in cellular proliferation and controls proliferation by suppressing cyclin D1 [54]. Luo et al. showed that miR-21 regulates β-catenin through the overexpression of the Sox2 protein, leading to cellular invasion and migration [55,56].

In their study, Masliantsev et al. [57] emphasized the critical role of the Hippo signaling pathway in regulating proliferation and inhibiting apoptosis through its influence on YAP/TAZ-TEAD (Figure 4). This highly conserved pathway controls multiple downstream oncogenic proteins, posing challenges for targeted drug interventions. Therefore, the Hippo signaling pathway holds promise as an important therapeutic target. One of the most well-known inhibitors of the Hippo pathway is a benzoporphyrin molecule called Verteporfin. Our KEGG pathway analysis identified the Hippo signaling pathway as the primary target for miR-21 (Figure 2A). Further investigations are needed to deepen our understanding of this association and explore potential therapeutic avenues involving miR-21.

### 4.2. miR-15b

The microRNA miR-15b has been well studied in various human tumors [58]. However, its role in cancer remains controversial, as it has been reported to be upregulated in certain cancers such as colorectal cancer [59], cervical cancer [60], and melanoma [61], while being downregulated in gastric cancer [62] and hepatocellular cancer [63]. 

Several studies have suggested that miR-15b is an onco-suppressive microRNA, as its downregulation is associated with the Karnofsky performance score and WHO glioma grade [58,64,65]. Additionally, Baraniskin et al. found that miR-15b was downregulated in cerebrospinal fluid samples of primary CNS lymphoma cases compared to the control group, with an AUC of 0.85 [26].

Bcl2, an oncogenic and antiapoptotic molecule, has been identified as a target of miR-15b [66,67,68,69]. The overexpression of miR-15b can arrest the cell cycle in G0/G1 phase or promote apoptosis in glioma cells [65]. Similarly, the increase in cyclin D1 due to miR-15b downregulation can improve cell proliferation [65,70], while cyclin D1 knockdown inhibits the proliferation and invasion of glioma cells by inducing apoptosis [71]. In glioma tissue, the concentration of Cripto-1 negatively correlates with miR-15b expression [72]. Cripto-1 is an oncogenic molecule that promotes the invasion of glioma cells and enhances the expression levels of MMP2 and MMP9 [72]. NRP-2, a receptor for VEGF, is also a target of miR-15b and mediates neuronal guidance, angiogenesis, and tumor progression [73,74,75]. The overexpression of miR-15b can inhibit angiogenesis via NRP-2 downregulation. Finally, SALL-4, a zinc-finger transcription factor important in gliomagenesis, is targeted by miR-15b, miR-195, and miR-103, which suppress glioma [64,76]. Xia et al. found that miR-15b played an onco-suppressive role by arresting cancerous cells at the G1/G0 phase via targeting the CCNE1 gene, which encodes cyclin E1, despite its upregulation in glioma [62].

Furthermore, KEGG pathway analysis of miR-15b revealed the involvement of other well-known pathways associated with cancer. The AMP-activated protein kinase (AMPK) pathway, a cellular energy sensor, has been identified as a significant target for miR-15b (Figure 4). Previous reports have suggested that AMPK exerts regulatory effects on GBM cell viability and lymphangiogenesis [77,78]. We found a significant association between miR-15b and TGF-β, similar to previous studies. TGF-β is a strong tumor suppressor in normal cells by increasing resistance to mitogen proliferative signals. Even in the primary stages of malignancy, this has an apoptotic effect [79]. However, in the tumoral environment, it plays a strong oncogenic role by inducing proliferation, angiogenesis, invasion, and immunosuppression (Figure 4). Previous reports have considered TGF-β as a therapeutic target [80]. Additionally, the FOXO pathway, known for its tumor suppressor activity in both glioma [81] and non-Hodgkin lymphomas [82], and the WNT pathway, associated with oncogenic activity [80], were identified as potential targets of miR-15b (Figure 2B and Figure 4).

Contrary to previous reports, there have been reports of miR-15b upregulation in glioma [62,83] and especially in GBM cases [84,85]. Our review of the literature showed that miR-15b concentration was higher in glioma than in PCNSL cases in both CSF [27] and plasma samples [22] (Figure 3). High expression of miR-15b was a statistically significant risk factor for poor survival in glioma patients [83]. The role of miR-15 in tumorigenesis and its levels in different tumors are not yet fully understood, necessitating further research. The variations in the reported findings may be attributed to differences in the methodologies employed and could potentially be influenced by diverse tumor microenvironments as we saw for TGF-β. 

### 4.3. miR-16

The microRNA miR-16 has been widely studied in cancer development, and it is commonly downregulated in various solid tumors [86] including glioma cells [87]. D’urso et al. reported a significant decrease in the plasma concentration of miR-16 in glioma compared with PCNSL and control cases. The downregulation of miR-16 is more pronounced in glioma than in PCNSL [22] (Figure 3), and its suppression directly correlates with the degree of malignancy [88].

Previous studies have indicated different roles for miR-16 in several cancers, such as bladder cancer [89], pancreatic cancer [90], thyroid cancer [91], colorectal cancer [92], and glioma. This study revealed that miR-16 targets the Hippo and P53 signaling pathways (Figure 2C); miR-16 is clustered with miR-15b at the 13q14 loci and targets different oncogenic molecules, including BCL2, MCL1, CCND1, and WNT3A [93]. 

Apoptosis is affected by miR-16 through the Bax/Bcl2 pathway. Bcl2 is an oncogenic and antiapoptotic mitochondrial protein involved in glioma development [66]. Thus, miR-16 inhibits Bcl2 mRNA translation and facilitates apoptosis. Through this mechanism, miR-16 overexpression inhibits U118 cell proliferation [87]. The upregulation of miR-16 inhibits the growth of hepatocellular cancer cells [92] and glioma cells [87] through the PI3K/AKT/mTOR signaling pathway (Figure 4).

The level of miR-16 inversely correlates with NF-κB 1 protein expression, and miR-16 directly reduces the expression of the NF-κB 1 protein, inhibiting glioma cell invasion through the NF-κB/MMP9 signaling pathway [88]. The expression of MMPs, especially MMP2, and MMP9, is upregulated and correlated with progression, tumor aggressiveness, and poor prognosis in glioma [94,95]. Dimerization of the NF-κB transcription factor at the κB sequence in the MMP9 promoter initiates MMP9 transcription, thus upregulating MMP9 protein expression [96].

### 4.4. miR-301a

The microRNA miR-301a is a well-known oncogene, which is upregulated in various cancers and promotes metastasis and proliferation [97,98,99]. In osteosarcoma cells, miR-301a may cause doxorubicin resistance by modulating the AMP-activated protein kinase alpha-1 pathway [100]. In pancreatic cancer cells, miR-301a acts as an NF-κB activator [101]. In breast cancer, miR-301a promotes tumor cell metastasis by inactivating PTEN and activating the WNT/β-catenin (Figure 4) pathway [98]. In pancreatic cancer, miR-301a may act as an oncogene by negatively regulating SAMD4 as its target [102], while in prostate cancer, miR-301a may be involved in metastasis through AR/TGF-β1/Smad/MMP9 signaling modulation [103]. In lung cancer, miR-301 has been reported to target MEOX2, while in breast cancer, it targets COL2A1, PTEN, BBC3, and FOXF2 [104,105]. Figure 2D shows other involved pathways of miR-301a such as PI3K/AKT. Previous reports have highlighted the association of miR-301a with prognosis and its correlation with radiation resistance mediated through the WNT pathway [24,57]. Lan et al. [24] reported that serum exosomes from GBM patients can increase the proliferation and invasion of H4 cells in vitro. The serum exosomal miR-301a level in glioma patients is significantly higher than that in patients with PCNSL and healthy controls. However, the miR-301a level is higher in PCNSL patients than in healthy controls (Figure 3). Nevertheless, considering the small sample size of PCNSL patients (n = 7), more data may be necessary to confirm this observation [24].

### 4.5. miR-711

The expression level of miR-711 varies in different cancers, acting as an oncogene in breast cancer and a tumor suppressor in cutaneous T-cell lymphoma and gastric cancer [106,107,108,109]. However, it is downregulated in prostate cancer [110]. While miR-711 is downregulated in PCNSL cases compared to GBM cases with an 8.77 fold change (*p* < 0.001), it is also downregulated in PCNSL cases compared to both glioma and normal cases [21]. To differentiate PCNSL and GBM cases, Drusco et al. [21] examined the levels of miR-451, miR-711, and miR-935. The control group showed the lowest level of miR-451 and moderate levels of miR-711 and miR-935. The PCNSL cases demonstrated a low level of miR-451, a lower level of miR-711, and an absence of miR-935. In contrast, the GBM cases revealed moderate expression for miR-451 and miR-711 and a complete absence of miR-935 [21] (Figure 3). 

Target prediction analysis revealed several cellular target genes for miR-711 [110]. Examples of validated targets include SP1, CDK4, AKT, and IRS1, while examples of predicted targets include skI, cyp2w1, CDK4, DNM2, ILK, ZNFR3, TXNIP, FADS1 VSIG2, CLCN5, and ALDH9A1, which are all involved in cancer progression [110]. Sabirzhanov et al. found that the inhibition of miR-711 increases DNA repair and reduces radiation-related apoptosis [109]. Other miR-711 pathways and targets of miR-711 are demonstrated in Figure 2E. The RAS/MAPK signaling pathway (Figure 4) has emerged as the most prominent pathway in our KEGG analysis, warranting further in-depth investigation in future studies. 

### 4.6. miR-205

The microRNA miR-205 is a potential diagnostic biomarker in various cancers, with its expression pattern and role varying according to different cancers [111]. In glioma [25], breast cancer [112], esophageal cancer [113], prostate cancer [114], and osteosarcoma [115], miR-205 concentration increases, leading to an inhibition of proliferation and invasion. However, in bladder cancer [116], ovarian cancer [117], and endometrial cancer [118], its concentration decreases, facilitating tumor initiation and proliferation. Overall, miR-205 is downregulated in most glioma cases, and this suppression directly correlates with glioma progression, malignancy, and inversely with the Karnofsky performance score of patients [25,119,120]. The overexpression of miR-205 inhibits glioma cells’ invasive behavior and improves apoptosis and cell-cycle arrest at the G1/G0 phase by targeting vascular endothelial growth factor A (VEGF A) (Figure 2F and Figure 4F) [119], which is the most potent mediator of angiogenesis in glioma [121]. ERBB3 overexpression in GBM cells is caused by promoter methylation or miR-205 downregulation as an onco-suppressor (Figure 4) [122], resulting in a metabolic increase and PI3k/AKT/mTOR pathway hyperactivation [123] (Figure 2F). Ji et al. revealed YAP1 as a target of miR-205 in glioma cells. YAP1 is part of the Hippo signaling pathway and plays a crucial role in cell proliferation, invasion, epithelial–mesenchymal transition, metastasis, differentiation, and survival [124] (Figure 2F). Although miR-205 is not studied in PCNSL as extensively as in glioma, Yue et al. reported the downregulation of serum miR-205 in PCNSL compared to healthy controls, with the miR-205 serum level in PCNSL cases being more pronounced than in glioma cases [25]. 

## 5. Conclusions

In conclusion, this review highlights the potential of circulating miRs as diagnostic biomarkers for differentiating glioma, including GBM, and PCNSL. Based on these findings, a combination of six miRs with distinct expression patterns could represent a promising approach to achieve accurate and timely diagnosis (Figure 3). Furthermore, exploring the use of circulating miRs in blood and CSF as diagnostic markers could provide valuable information for patient stratification and better clinical management. However, more research is necessary to evaluate the diagnostic efficacy of these miRs and their ability to predict patient prognosis. Additionally, validation studies in larger patient cohorts and across different populations are needed to establish the clinical utility of these miRs in routine clinical practice. Overall, the identification and validation of circulating miRs as biomarkers for glioma and PCNSL could significantly improve patient outcomes and quality of life through earlier diagnosis and appropriate intervention.

## Figures and Tables

**Table 1:** Summary of the included studies.

Article	Method	Sample	Study Groups	MicroRNA Profile	Change Pattern	Assessments
**Mao** **2014** [19]	qRT-PCR	Serum/Plasma	GBM: 32 HC: 47 PCNSL: 56	miR-21	HC < GBM, *p* < 0.0001 HC < PCNSL, *p* < 0.0001 GBM < PCNSL, *p* < 0.0001	**GBM vs. PCNSL AUC (CI)** 0.883 (0.813–0.954), *p* < 0.0001
	qRT-PCR	Serum/Plasma	GBM: 23 HC: 35 PCNSL: 37	miR-21	HC < GBM, *p* < 0.0001 HC < PCNSL, *p* < 0.0001 GBM < PCNSL, *p* < 0.0001	**GBM vs. PCNSL AUC (CI)** 0.851 (0.755–0.947)
**Yang** **2019** [20]	RT-qPCR	Serum/Plasma	GBM: 25 HC: 25 PCNSL: 25	miR-21	HC < PCNSL, *p* < 0.05 GBM < PCNSL, *p* < 0.05 HC < GBM	-
**Drusco** **2015** [21]	RT-PCR	CSF	GBM: 4 (17 sample) PCNSL: 3 HC: 14 Glioma: 9	miR-451 miR-711 miR-935 miR-125b miR-223	HC < PCNSL < glioma PCNSL: downregulated < (glioma, Normal) PCNSL < GBM Not expressed in glioma and PCNSL < Normal HC < PCNSL < glioma HC < PCNSL < glioma	**miR Concentration (HC/Glioma/PCNSL)** miR-451: 4.20 ± 2.55/11.75 ± 5.74/10.23 ± 6.32 miR-711: 8.78 ± 4.97/9.54 ± 0.61/6.40 ± 0.53 miR-935: 10.25 ± 4.74/0/0 miR-125b: 3.89 ± 0.92/9.72 ± 2.42/7.82 ± 2.56 miR-223: 6.34 ± 5.48/13.24 ± 1.89/8.86 ± 0.97 **Fold change (GBM/PCNSL):** miR-711: 8.77, *p* < 0.001
**D’urso** **2015** [22]	qRT-PCR	Serum/Plasma	GBM: 16 PCNSL: 36 VND: 30	miR-16	GBM < PCNSL < VND	-
	qRT-PCR	Serum/Plasma	Glioma: 30 PCNSL: 36 VND: 30	miR-15 bmiR-21 miR-16	VND < PCNSL < Glioma VND < Glioma < PCNSL Glioma < PCNSL < VND	-
**Si** **2022** [23]	-	Serum/Plasma	Glioma: 170 PCNSL: 42	miR-6820-3p miR-6803-3p miR-4756-5p miR-30a-3p miR-548am-3p miR-487a-3p miR-3918 miR-4751 miR-371a-3p miR-146a-3p	Glioma < PCNSL, *p* = 0.00028 Glioma < PCNSL, *p* = 1.4 × 10^−5^ Glioma < PCNSL, *p* = 0.00068 Glioma < PCNSL, *p* = 1.9 × 10^−6^ Glioma < PCNSL, *p* = 0.0053 Glioma < PCNSL, *p* = 0.0003 Glioma < PCNSL, *p* = 0.00027 Glioma < PCNSL, *p* = 0.00011 Glioma < PCNSL, *p* = 0.0007 Glioma < PCNSL, *p* = 0.0017	**PCNSL vs. Glioma AUC (CI)** 0.681 (0.579–0.774) 0.716 (0.646–0.786) 0.669 (0.578–0.755) 0.737 (0.660–0.810) 0.639 (0.531–0.743) 0.681 (0.593–0.762) 0.682 (0.586–0.766) 0.693 (0.602–0.778) 0.669 (0.578–0.757) 0.656 (0.551–0.754)
**Lan** **2018** [24]	qRT-PCR	Serum/Plasma	Glioma: 59 PCNSL: 7 HC: 28	miR-301a	HC < Glioma *p* < 0.01 PCNSL < Glioma *p* < 0.01 HC < PCNSL	-
**Yue** **2016** [25]	qRT-PCR	Serum/Plasma	Glioma: 30 PCNSL: 6 HC: 20	miR-205	Glioma < PCNSL, *p* < 0.01 Glioma < HC, *p* < 0.01 Glioma < PCNSL < HC	-
**Baraniskin** **2012** [26]	qRT-PCR	CSF	Glioma: 10 PCNSL: 23 VND: 10	miR-21 miR-15b	Control < Glioma < PCNSL, *p* < 0.05 Control < PCNSL < Glioma, *p* < 0.05	-

Note: miR: microRNA, VND: various neurological disorders, HC: healthy control, PCNSL: primary CNS lymphoma.

## Data Availability

Data are available upon request.

## Supplementary Materials

The following supporting information can be downloaded at: https://www.mdpi.com/article/10.3390/cancers15143628/s1, Table S1. Search string.

## References

[1] Surawicz T.S., McCarthy B.J., Kupelian V., Jukich P.J., Bruner J.M., Davis F.G. (1999). Descriptive epidemiology of primary brain and CNS tumors: Results from the Central Brain Tumor Registry of the United States, 1990–1994. Neuro-Oncol..

[2] Posti J.P., Bori M., Kauko T., Sankinen M., Nordberg J., Rahi M., Frantzén J., Vuorinen V., Sipilä J.O. (2015). Presenting symptoms of glioma in adults. Acta Neurol. Scand..

[3] Ostrom Q.T., Bauchet L., Davis F.G., Deltour I., Fisher J.L., Langer C.E., Pekmezci M., Schwartzbaum J.A., Turner M.C., Walsh K.M. (2014). The epidemiology of glioma in adults: A “state of the science” review. Neuro-Oncol..

[4] Kim Y.Z., Kim C.Y., Lim D.H. (2022). The Overview of Practical Guidelines for Gliomas by KSNO, NCCN, and EANO. Brain Tumor Res. Treat..

[5] Löw S., Han C.H., Batchelor T.T. (2018). Primary central nervous system lymphoma. Ther. Adv. Neurol. Disord..

[6] Saini J., Kumar Gupta P., Awasthi A., Pandey C.M., Singh A., Patir R., Ahlawat S., Sadashiva N., Mahadevan A., Kumar Gupta R. (2018). Multiparametric imaging-based differentiation of lymphoma and glioblastoma: Using T1-perfusion, diffusion, and susceptibility-weighted MRI. Clin. Radiol..

[7] Lee J.Y., Bjørnerud A., Park J.E., Lee B.E., Kim J.H., Kim H.S. (2019). Permeability measurement using dynamic susceptibility contrast magnetic resonance imaging enhances differential diagnosis of primary central nervous system lymphoma from glioblastoma. Eur. Radiol..

[8] Wolburg H., Noell S., Fallier-Becker P., Mack A.F., Wolburg-Buchholz K. (2012). The disturbed blood-brain barrier in human glioblastoma. Mol. Asp. Med..

[9] Baraniskin A., Kuhnhenn J., Schlegel U., Schmiegel W., Hahn S., Schroers R. (2012). MicroRNAs in cerebrospinal fluid as biomarker for disease course monitoring in primary central nervous system lymphoma. J. Neuro-Oncol..

[10] Zajdel M., Rymkiewicz G., Sromek M., Cieslikowska M., Swoboda P., Kulinczak M., Goryca K., Bystydzienski Z., Blachnio K., Ostrowska B. (2019). Tumor and Cerebrospinal Fluid microRNAs in Primary Central Nervous System Lymphomas. Cancers.

[11] Di Leva G., Croce C.M. (2013). miRNA profiling of cancer. Curr. Opin. Genet. Dev..

[12] Salarinia R., Sahebkar A., Peyvandi M., Mirzaei H.R., Jaafari M.R., Riahi M.M., Ebrahimnejad H., Nahand J.S., Hadjati J., Asrami M.O. (2016). Epi-Drugs and Epi-miRs: Moving Beyond Current Cancer Therapies. Curr. Cancer Drug Targets.

[13] Qiu S., Lin S., Hu D., Feng Y., Tan Y., Peng Y. (2013). Interactions of miR-323/miR-326/miR-329 and miR-130a/miR-155/miR-210 as prognostic indicators for clinical outcome of glioblastoma patients. J. Transl. Med..

[14] Gareev I., Beylerli O., Liang Y., Xiang H., Liu C., Xu X., Yuan C., Ahmad A., Yang G. (2021). The Role of MicroRNAs in Therapeutic Resistance of Malignant Primary Brain Tumors. Front. Cell Dev. Biol..

[15] Zhang S., Wan Y., Pan T., Gu X., Qian C., Sun G., Sun L., Xiang Y., Wang Z., Shi L. (2012). MicroRNA-21 inhibitor sensitizes human glioblastoma U251 stem cells to chemotherapeutic drug temozolomide. J. Mol. Neurosci. MN.

[16] Diaz L.A., Bardelli A. (2014). Liquid biopsies: Genotyping circulating tumor DNA. J. Clin. Oncol..

[17] Ilié M., Hofman P. (2016). Pros: Can tissue biopsy be replaced by liquid biopsy?. Transl. Lung Cancer Res..

[18] Page M.J., McKenzie J.E., Bossuyt P.M., Boutron I., Hoffmann T.C., Mulrow C.D., Shamseer L., Tetzlaff J.M., Akl E.A., Brennan S.E. (2021). The PRISMA 2020 statement: An updated guideline for reporting systematic reviews. BMJ.

[19] Mao X., Sun Y., Tang J. (2014). Serum miR-21 is a diagnostic and prognostic marker of primary central nervous system lymphoma. Neurol. Sci..

[20] Yang K., Wang S., Cheng Y., Tian Y., Hou J. (2019). Role of miRNA-21 in the diagnosis and prediction of treatment efficacy of primary central nervous system lymphoma. Oncol. Lett..

[21] Si P.P., Zhou X.H., Qu Z.Z. (2022). Identification of Serum miRNAs as Effective Diagnostic Biomarkers for Distinguishing Primary Central Nervous System Lymphoma from Glioma. J. Immunol. Res..

[22] Lan F., Qing Q., Pan Q., Hu M., Yu H., Yue X. (2018). Serum exosomal miR-301a as a potential diagnostic and prognostic biomarker for human glioma. Cell. Oncol..

[23] Yue X., Lan F., Hu M., Pan Q., Wang Q., Wang J. (2016). Downregulation of serum microRNA-205 as a potential diagnostic and prognostic biomarker for human glioma. J. Neurosurg..

[24] Ivo D’Urso P., Fernando D’Urso O., Damiano Gianfreda C., Mezzolla V., Storelli C., Marsigliante S. (2015). miR-15b and miR-21 as Circulating Biomarkers for Diagnosis of Glioma. Curr. Genom..

[25] Baraniskin A., Kuhnhenn J., Schlegel U., Maghnouj A., Zöllner H., Schmiegel W., Hahn S., Schroers R. (2012). Identification of microRNAs in the cerebrospinal fluid as biomarker for the diagnosis of glioma. Neuro Oncol..

[26] Drusco A., Bottoni A., Laganà A., Acunzo M., Fassan M., Cascione L., Antenucci A., Kumchala P., Vicentini C., Gardiman M.P. (2015). A differentially expressed set of microRNAs in cerebro-spinal fluid (CSF) can diagnose CNS malignancies. Oncotarget.

[27] Baraniskin A., Kuhnhenn J., Schlegel U., Chan A., Deckert M., Gold R., Maghnouj A., Zöllner H., Reinacher-Schick A., Schmiegel W. (2011). Identification of microRNAs in the cerebrospinal fluid as marker for primary diffuse large B-cell lymphoma of the central nervous system. Blood.

[28] Medina P.P., Nolde M., Slack F.J. (2010). OncomiR addiction in an in vivo model of microRNA-21-induced pre-B-cell lymphoma. Nature.

[29] Wei D., Wan Q., Li L., Jin H., Liu Y., Wang Y., Zhang G. (2015). MicroRNAs as Potential Biomarkers for Diagnosing Cancers of Central Nervous System: A Meta-analysis. Mol. Neurobiol..

[30] Conti A., Aguennouz M., La Torre D., Tomasello C., Cardali S., Angileri F.F., Maio F., Cama A., Germanò A., Vita G. (2009). miR-21 and 221 upregulation and miR-181b downregulation in human grade II-IV astrocytic tumors. J. Neuro-Oncol..

[31] Chan J.A., Krichevsky A.M., Kosik K.S. (2005). MicroRNA-21 is an antiapoptotic factor in human glioblastoma cells. Cancer Res..

[32] Chen Y., Liu W., Chao T., Zhang Y., Yan X., Gong Y., Qiang B., Yuan J., Sun M., Peng X. (2008). MicroRNA-21 down-regulates the expression of tumor suppressor PDCD4 in human glioblastoma cell T98G. Cancer Lett..

[33] Zhou J., Wang K.C., Wu W., Subramaniam S., Shyy J.Y., Chiu J.J., Li J.Y., Chien S. (2011). MicroRNA-21 targets peroxisome proliferators-activated receptor-alpha in an autoregulatory loop to modulate flow-induced endothelial inflammation. Proc. Natl. Acad. Sci. USA.

[34] Zhou X., Ren Y., Moore L., Mei M., You Y., Xu P., Wang B., Wang G., Jia Z., Pu P. (2010). Downregulation of miR-21 inhibits EGFR pathway and suppresses the growth of human glioblastoma cells independent of PTEN status. Lab. Investig..

[35] Wu W., Sun M., Zou G.M., Chen J. (2007). MicroRNA and cancer: Current status and prospective. Int. J. Cancer.

[36] Papagiannakopoulos T., Shapiro A., Kosik K.S. (2008). MicroRNA-21 targets a network of key tumor-suppressive pathways in glioblastoma cells. Cancer Res..

[37] Yu J., Cai X., He J., Zhao W., Wang Q., Liu B. (2013). Microarray-based analysis of gene regulation by transcription factors and microRNAs in glioma. Neurol. Sci..

[38] Shi R., Wang P.Y., Li X.Y., Chen J.X., Li Y., Zhang X.Z., Zhang C.G., Jiang T., Li W.B., Ding W. (2015). Exosomal levels of miRNA-21 from cerebrospinal fluids associated with poor prognosis and tumor recurrence of glioma patients. Oncotarget.

[39] Seo Y.E., Suh H.W., Bahal R., Josowitz A., Zhang J., Song E., Cui J., Noorbakhsh S., Jackson C., Bu T. (2019). Nanoparticle-mediated intratumoral inhibition of miR-21 for improved survival in glioblastoma. Biomaterials.

[40] Abels E.R., Maas S.L.N., Nieland L., Wei Z., Cheah P.S., Tai E., Kolsteeg C.J., Dusoswa S.A., Ting D.T., Hickman S. (2019). Glioblastoma-Associated Microglia Reprogramming Is Mediated by Functional Transfer of Extracellular miR-21. Cell Rep..

[41] Gaur A.B., Holbeck S.L., Colburn N.H., Israel M.A. (2011). Downregulation of Pdcd4 by mir-21 facilitates glioblastoma proliferation in vivo. Neuro Oncol..

[42] Faragalla H., Youssef Y.M., Scorilas A., Khalil B., White N.M., Mejia-Guerrero S., Khella H., Jewett M.A., Evans A., Lichner Z. (2012). The clinical utility of miR-21 as a diagnostic and prognostic marker for renal cell carcinoma. J. Mol. Diagn..

[43] Chai C., Song L.J., Han S.Y., Li X.Q., Li M. (2018). MicroRNA-21 promotes glioma cell proliferation and inhibits senescence and apoptosis by targeting SPRY1 via the PTEN/PI3K/AKT signaling pathway. CNS Neurosci. Ther..

[44] Han L., Yue X., Zhou X., Lan F.M., You G., Zhang W., Zhang K.L., Zhang C.Z., Cheng J.Q., Yu S.Z. (2012). MicroRNA-21 expression is regulated by β-catenin/STAT3 pathway and promotes glioma cell invasion by direct targeting RECK. CNS Neurosci. Ther..

[45] Gabriely G., Wurdinger T., Kesari S., Esau C.C., Burchard J., Linsley P.S., Krichevsky A.M. (2008). MicroRNA 21 promotes glioma invasion by targeting matrix metalloproteinase regulators. Mol. Cell Biol..

[46] Li Y., Li W., Yang Y., Lu Y., He C., Hu G., Liu H., Chen J., He J., Yu H. (2009). MicroRNA-21 targets LRRFIP1 and contributes to VM-26 resistance in glioblastoma multiforme. Brain Res..

[47] Ho K.G., Grommes C. (2019). Molecular profiling of primary central nervous system lymphomas—Predictive and prognostic value?. Curr. Opin. Neurol..

[48] Geng H., Gao H., Kadoch C., Lu M., Chen L., Anjum R., Drew L., Degorce S., Dillman K., Mayo M. (2016). Targeting NF-KB Activation in Novel Intracranial Models of CNS Lymphoma. Blood.

[49] Kwak H.J., Kim Y.J., Chun K.R., Woo Y.M., Park S.J., Jeong J.A., Jo S.H., Kim T.H., Min H.S., Chae J.S. (2011). Downregulation of Spry2 by miR-21 triggers malignancy in human gliomas. Oncogene.

[50] Lan F., Pan Q., Yu H., Yue X. (2015). Sulforaphane enhances temozolomide-induced apoptosis because of down-regulation of miR-21 via Wnt/β-catenin signaling in glioblastoma. J. Neurochem..

[51] Huang K., Zhang J.X., Han L., You Y.P., Jiang T., Pu P.Y., Kang C.S. (2010). MicroRNA roles in beta-catenin pathway. Mol. Cancer.

[52] Frenquelli M., Tonon G. (2020). WNT Signaling in Hematological Malignancies. Front. Oncol..

[53] Nager M., Bhardwaj D., Cantí C., Medina L., Nogués P., Herreros J. (2012). β-Catenin Signalling in Glioblastoma Multiforme and Glioma-Initiating Cells. Chemother. Res. Pract..

[54] Farioli-Vecchioli S., Tanori M., Micheli L., Mancuso M., Leonardi L., Saran A., Ciotti M.T., Ferretti E., Gulino A., Pazzaglia S. (2007). Inhibition of medulloblastoma tumorigenesis by the antiproliferative and pro-differentiative gene PC3. Faseb J..

[55] Sathyan P., Zinn P.O., Marisetty A.L., Liu B., Kamal M.M., Singh S.K., Bady P., Lu L., Wani K.M., Veo B.L. (2015). Mir-21-Sox2 Axis Delineates Glioblastoma Subtypes with Prognostic Impact. J. Neurosci..

[56] Luo G., Luo W., Sun X., Lin J., Wang M., Zhang Y., Luo W., Zhang Y. (2017). MicroRNA-21 promotes migration and invasion of glioma cells via activation of Sox2 and β-catenin signaling. Mol. Med. Rep..

[57] Masliantsev K., Karayan-Tapon L., Guichet P.O. (2021). Hippo Signaling Pathway in Gliomas. Cells.

[58] Sun G., Yan S., Shi L., Wan Z., Jiang N., Li M., Guo J. (2015). Decreased Expression of miR-15b in Human Gliomas is Associated with Poor Prognosis. Cancer Biother. Radiopharm..

[59] Xi Y., Formentini A., Chien M., Weir D.B., Russo J.J., Ju J., Kornmann M., Ju J. (2006). Prognostic Values of microRNAs in Colorectal Cancer. Biomark. Insights.

[60] Wang X., Tang S., Le S.Y., Lu R., Rader J.S., Meyers C., Zheng Z.M. (2008). Aberrant expression of oncogenic and tumor-suppressive microRNAs in cervical cancer is required for cancer cell growth. PLoS ONE.

[61] Satzger I., Mattern A., Kuettler U., Weinspach D., Voelker B., Kapp A., Gutzmer R. (2010). MicroRNA-15b represents an independent prognostic parameter and is correlated with tumor cell proliferation and apoptosis in malignant melanoma. Int. J. Cancer.

[62] Xia L., Zhang D., Du R., Pan Y., Zhao L., Sun S., Hong L., Liu J., Fan D. (2008). miR-15b and miR-16 modulate multidrug resistance by targeting BCL2 in human gastric cancer cells. Int. J. Cancer.

[63] Chung G.E., Yoon J.H., Myung S.J., Lee J.H., Lee S.H., Lee S.M., Kim S.J., Hwang S.Y., Lee H.S., Kim C.Y. (2010). High expression of microRNA-15b predicts a low risk of tumor recurrence following curative resection of hepatocellular carcinoma. Oncol. Rep..

[64] Chen L.P., Zhang N.N., Ren X.Q., He J., Li Y. (2018). miR-103/miR-195/miR-15b Regulate SALL4 and Inhibit Proliferation and Migration in Glioma. Molecules.

[65] Sun G., Shi L., Yan S., Wan Z., Jiang N., Fu L., Li M., Guo J. (2014). MiR-15b targets cyclin D1 to regulate proliferation and apoptosis in glioma cells. Biomed. Res. Int..

[66] Rodriguez-Pereira C., Suarez-Peñaranda J.M., Barros F., Sobrido M.J., Vazquez-Salvado M., Forteza J. (2001). Analysis of 2 antiapoptotic factors in gliomas: Bcl-2 overexpression and p53 mutations. Arch. Pathol. Lab. Med..

[67] Murakami Y., Yasuda T., Saigo K., Urashima T., Toyoda H., Okanoue T., Shimotohno K. (2006). Comprehensive analysis of microRNA expression patterns in hepatocellular carcinoma and non-tumorous tissues. Oncogene.

[68] Hayashita Y., Osada H., Tatematsu Y., Yamada H., Yanagisawa K., Tomida S., Yatabe Y., Kawahara K., Sekido Y., Takahashi T. (2005). A polycistronic microRNA cluster, miR-17-92, is overexpressed in human lung cancers and enhances cell proliferation. Cancer Res..

[69] Yanaihara N., Caplen N., Bowman E., Seike M., Kumamoto K., Yi M., Stephens R.M., Okamoto A., Yokota J., Tanaka T. (2006). Unique microRNA molecular profiles in lung cancer diagnosis and prognosis. Cancer Cell.

[70] Tashiro E., Tsuchiya A., Imoto M. (2007). Functions of cyclin D1 as an oncogene and regulation of cyclin D1 expression. Cancer Sci..

[71] Wang J., Wang Q., Cui Y., Liu Z.Y., Zhao W., Wang C.L., Dong Y., Hou L., Hu G., Luo C. (2012). Knockdown of cyclin D1 inhibits proliferation, induces apoptosis, and attenuates the invasive capacity of human glioblastoma cells. J. Neurooncol..

[72] Sun G., Yan S.S., Shi L., Wan Z.Q., Jiang N., Fu L.S., Li M., Guo J. (2016). MicroRNA-15b suppresses the growth and invasion of glioma cells through targeted inhibition of cripto-1 expression. Mol. Med. Rep..

[73] Zheng X., Chopp M., Lu Y., Buller B., Jiang F. (2013). MiR-15b and miR-152 reduce glioma cell invasion and angiogenesis via NRP-2 and MMP-3. Cancer Lett..

[74] Geretti E., Klagsbrun M. (2007). Neuropilins: Novel targets for anti-angiogenesis therapies. Cell Adh Migr..

[75] Seyedmirzaei H., Shobeiri P., Turgut M., Hanaei S., Rezaei N. (2021). VEGF levels in patients with glioma: A systematic review and meta-analysis. Rev. Neurosci..

[76] He J., Zhang W., Zhou Q., Zhao T., Song Y., Chai L., Li Y. (2013). Low-expression of microRNA-107 inhibits cell apoptosis in glioma by upregulation of SALL4. Int. J. Biochem. Cell Biol..

[77] Chhipa R.R., Fan Q., Anderson J., Muraleedharan R., Huang Y., Ciraolo G., Chen X., Waclaw R., Chow L.M., Khuchua Z. (2018). AMP kinase promotes glioblastoma bioenergetics and tumour growth. Nat. Cell Biol..

[78] Hoffman A.E., Demanelis K., Fu A., Zheng T., Zhu Y. (2013). Association of AMP-activated protein kinase with risk and progression of non-Hodgkin lymphoma. Cancer Epidemiol. Biomark. Prev. A Publ. Am. Assoc. Cancer Res. Cosponsored Am. Soc. Prev. Oncol..

[79] Han J., Alvarez-Breckenridge C.A., Wang Q.E., Yu J. (2015). TGF-β signaling and its targeting for glioma treatment. Am. J. Cancer Res..

[80] Latour M., Her N.G., Kesari S., Nurmemmedov E. (2021). WNT Signaling as a Therapeutic Target for Glioblastoma. Int. J. Mol. Sci..

[81] Yan H., Wu A. (2018). FOXO1 is crucial in glioblastoma cell tumorigenesis and regulates the expression of SIRT1 to suppress senescence in the brain. Mol. Med. Rep..

[82] Sablon A., Bollaert E., Pirson C., Velghe A.I., Demoulin J.B. (2022). FOXO1 forkhead domain mutants in B-cell lymphoma lack transcriptional activity. Sci. Rep..

[83] Pang C., Guan Y., Zhao K., Chen L., Bao Y., Cui R., Li G., Wang Y. (2015). Up-regulation of microRNA-15b correlates with unfavorable prognosis and malignant progression of human glioma. Int. J. Clin. Exp. Pathol..

[84] Guan Y., Mizoguchi M., Yoshimoto K., Hata N., Shono T., Suzuki S.O., Araki Y., Kuga D., Nakamizo A., Amano T. (2010). MiRNA-196 is upregulated in glioblastoma but not in anaplastic astrocytoma and has prognostic significance. Clin. Cancer Res..

[85] Malzkorn B., Wolter M., Liesenberg F., Grzendowski M., Stühler K., Meyer H.E., Reifenberger G. (2010). Identification and functional characterization of microRNAs involved in the malignant progression of gliomas. Brain Pathol..

[86] Calin G.A., Croce C.M. (2006). MicroRNA-cancer connection: The beginning of a new tale. Cancer Res..

[87] Yang Y., Zhao F. (2020). MicroRNA-16 inhibits the growth and metastasis of human glioma cells via modulation of PI3K/AKT/mTOR signalling pathway. Arch. Med. Sci..

[88] Yang T.Q., Lu X.J., Wu T.F., Ding D.D., Zhao Z.H., Chen G.L., Xie X.S., Li B., Wei Y.X., Guo L.C. (2014). MicroRNA-16 inhibits glioma cell growth and invasion through suppression of BCL2 and the nuclear factor-κB1/MMP9 signaling pathway. Cancer Sci..

[89] Yu Q., Liu P., Han G., Xue X., Ma D. (2020). CircRNA circPDSS1 promotes bladder cancer by down-regulating miR-16. Biosci. Rep..

[90] Gao L., He S.B., Li D.C. (2014). Effects of miR-16 plus CA19-9 detections on pancreatic cancer diagnostic performance. Clin. Lab..

[91] Xiong H., Yu H., Jia G., Yu J., Su Y., Zhang J., Zhou J. (2021). circZFR regulates thyroid cancer progression by the miR-16/MAPK1 axis. Env. Toxicol..

[92] Wu H., Wei M., Jiang X., Tan J., Xu W., Fan X., Zhang R., Ding C., Zhao F., Shao X. (2020). lncRNA PVT1 Promotes Tumorigenesis of Colorectal Cancer by Stabilizing miR-16-5p and Interacting with the VEGFA/VEGFR1/AKT Axis. Mol. Ther. Nucleic Acids.

[93] Aqeilan R.I., Calin G.A., Croce C.M. (2010). miR-15a and miR-16-1 in cancer: Discovery, function and future perspectives. Cell Death Differ..

[94] Kondraganti S., Mohanam S., Chintala S.K., Kin Y., Jasti S.L., Nirmala C., Lakka S.S., Adachi Y., Kyritsis A.P., Ali-Osman F. (2000). Selective suppression of matrix metalloproteinase-9 in human glioblastoma cells by antisense gene transfer impairs glioblastoma cell invasion. Cancer Res..

[95] Rao J.S. (2003). Molecular mechanisms of glioma invasiveness: The role of proteases. Nat. Rev. Cancer.

[96] Sato H., Seiki M. (1993). Regulatory mechanism of 92 kDa type IV collagenase gene expression which is associated with invasiveness of tumor cells. Oncogene.

[97] Yu H., Li H., Qian H., Jiao X., Zhu X., Jiang X., Dai G., Huang J. (2014). Upregulation of miR-301a correlates with poor prognosis in triple-negative breast cancer. Med. Oncol..

[98] Ma F., Zhang J., Zhong L., Wang L., Liu Y., Wang Y., Peng L., Guo B. (2014). Upregulated microRNA-301a in breast cancer promotes tumor metastasis by targeting PTEN and activating Wnt/β-catenin signaling. Gene.

[99] Wang M., Li C., Yu B., Su L., Li J., Ju J., Yu Y., Gu Q., Zhu Z., Liu B. (2013). Overexpressed miR-301a promotes cell proliferation and invasion by targeting RUNX3 in gastric cancer. J. Gastroenterol..

[100] Zhang Y., Duan G., Feng S. (2015). MicroRNA-301a modulates doxorubicin resistance in osteosarcoma cells by targeting AMP-activated protein kinase alpha 1. Biochem. Biophys. Res. Commun..

[101] Lu Z., Li Y., Takwi A., Li B., Zhang J., Conklin D.J., Young K.H., Martin R., Li Y. (2011). miR-301a as an NF-κB activator in pancreatic cancer cells. Embo J..

[102] Xia X., Zhang K., Cen G., Jiang T., Cao J., Huang K., Huang C., Zhao Q., Qiu Z. (2015). MicroRNA-301a-3p promotes pancreatic cancer progression via negative regulation of SMAD4. Oncotarget.

[103] Xie H., Li L., Zhu G., Dang Q., Ma Z., He D., Chang L., Song W., Chang H.C., Krolewski J.J. (2016). Correction: Infiltrated pre-adipocytes increase prostate cancer metastasis via modulation of the miR-301a/androgen receptor (AR)/TGF-β1/Smad/MMP9 signals. Oncotarget.

[104] Cao G., Huang B., Liu Z., Zhang J., Xu H., Xia W., Li J., Li S., Chen L., Ding H. (2010). Intronic miR-301 feedback regulates its host gene, ska2, in A549 cells by targeting MEOX2 to affect ERK/CREB pathways. Biochem. Biophys. Res. Commun..

[105] Shi W., Gerster K., Alajez N.M., Tsang J., Waldron L., Pintilie M., Hui A.B., Sykes J., P’ng C., Miller N. (2011). MicroRNA-301 mediates proliferation and invasion in human breast cancer. Cancer Res..

[106] Ralfkiaer U., Hagedorn P.H., Bangsgaard N., Løvendorf M.B., Ahler C.B., Svensson L., Kopp K.L., Vennegaard M.T., Lauenborg B., Zibert J.R. (2011). Diagnostic microRNA profiling in cutaneous T-cell lymphoma (CTCL). Blood.

[107] Liao A., Tan G., Chen L., Zhou W., Hu H. (2016). RASSF1A inhibits gastric cancer cell proliferation by miR-711- mediated downregulation of CDK4 expression. Oncotarget.

[108] Hu J.Y., Yi W., Zhang M.Y., Xu R., Zeng L.S., Long X.R., Zhou X.M., Zheng X.S., Kang Y., Wang H.Y. (2016). MicroRNA-711 is a prognostic factor for poor overall survival and has an oncogenic role in breast cancer. Oncol. Lett..

[109] Sabirzhanov B., Stoica B.A., Zhao Z., Loane D.J., Wu J., Dorsey S.G., Faden A.I. (2016). miR-711 upregulation induces neuronal cell death after traumatic brain injury. Cell Death Differ..

[110] Waseem M., Ahmad M.K., Srivatava V.K., Rastogi N., Serajuddin M., Kumar S., Mishra D.P., Sankhwar S.N., Mahdi A.A. (2017). Evaluation of miR-711 as Novel Biomarker in Prostate Cancer Progression. Asian Pac. J. Cancer Prev..

[111] Chao C.H., Chang C.C., Wu M.J., Ko H.W., Wang D., Hung M.C., Yang J.Y., Chang C.J. (2014). MicroRNA-205 signaling regulates mammary stem cell fate and tumorigenesis. J. Clin. Investig..

[112] Wang Z., Liao H., Deng Z., Yang P., Du N., Zhanng Y., Ren H. (2013). miRNA-205 affects infiltration and metastasis of breast cancer. Biochem. Biophys. Res. Commun..

[113] Xu H., Yao Y., Meng F., Qian X., Jiang X., Li X., Gao Z., Gao L. (2015). Predictive Value of Serum miR-10b, miR-29c, and miR-205 as Promising Biomarkers in Esophageal Squamous Cell Carcinoma Screening. Medicine.

[114] Verdoodt B., Neid M., Vogt M., Kuhn V., Liffers S.T., Palisaar R.J., Noldus J., Tannapfel A., Mirmohammadsadegh A. (2013). MicroRNA-205, a novel regulator of the anti-apoptotic protein Bcl2, is downregulated in prostate cancer. Int. J. Oncol..

[115] Yang G., Zhang P., Lv A., Liu Y., Wang G. (2016). MiR-205 functions as a tumor suppressor via targeting TGF-α in osteosarcoma. Exp. Mol. Pathol..

[116] Fang Z., Dai W., Wang X., Chen W., Shen C., Ye G., Li L. (2016). Circulating miR-205: A promising biomarker for the detection and prognosis evaluation of bladder cancer. Tumour Biol..

[117] Li J., Hu K., Gong G., Zhu D., Wang Y., Liu H., Wu X. (2017). Upregulation of MiR-205 transcriptionally suppresses SMAD4 and PTEN and contributes to human ovarian cancer progression. Sci. Rep..

[118] Jin C., Liang R. (2015). miR-205 promotes epithelial-mesenchymal transition by targeting AKT signaling in endometrial cancer cells. J. Obs. Gynaecol. Res..

[119] Yue X., Wang P., Xu J., Zhu Y., Sun G., Pang Q., Tao R. (2012). MicroRNA-205 functions as a tumor suppressor in human glioblastoma cells by targeting VEGF-A. Oncol. Rep..

[120] Hou S.X., Ding B.J., Li H.Z., Wang L., Xia F., Du F., Liu L.J., Liu Y.H., Liu X.D., Jia J.F. (2013). Identification of microRNA-205 as a potential prognostic indicator for human glioma. J. Clin. Neurosci..

[121] Kim K.J., Li B., Winer J., Armanini M., Gillett N., Phillips H.S., Ferrara N. (1993). Inhibition of vascular endothelial growth factor-induced angiogenesis suppresses tumour growth in vivo. Nature.

[122] De Bacco F., Orzan F., Erriquez J., Casanova E., Barault L., Albano R., D’Ambrosio A., Bigatto V., Reato G., Patanè M. (2021). ERBB3 overexpression due to miR-205 inactivation confers sensitivity to FGF, metabolic activation, and liability to ERBB3 targeting in glioblastoma. Cell Rep..

[123] Goncalves M.D., Hopkins B.D., Cantley L.C. (2018). Phosphatidylinositol 3-Kinase, Growth Disorders, and Cancer. N. Engl. J. Med..

[124] Zeng Q., Hong W. (2008). The emerging role of the hippo pathway in cell contact inhibition, organ size control, and cancer development in mammals. Cancer Cell.

