# Supplementary material for: Micro RNAs as a Diagnostic Marker between Glioma and Primary CNS Lymphoma: A Systematic Review
Source: Cancers (Basel). 2023 Jul 14;15(14):3628. doi: 10.3390/cancers15143628 (PMC10377645; doi:10.3390/cancers15143628)
Supplement: Supplementary file 1 [file cancers-15-03628-s001.zip › cancers-2376803-supplementary.pdf]

Table S1. Search string.

Database	Search strategy
PubMed	(microRNA*[TIAB] OR miRNA*[TIAB] OR mir[TIAB] OR micro-RNA[TIAB] OR "Micro RNA"[TIAB] OR stRNA[TIAB] OR "Temporal RNA"[TIAB] OR Pre-miRNA[TIAB] OR Pri-miRNA[TIAB] OR "MicroRNAs"[Mesh]) AND ("primary central nervous system lymphoma"[TIAB] OR "CNS DLBCL"[TIAB] OR PCNSL[TIAB] OR "central nervous system lymphoma"[TIAB] OR "primary CNS lymphoma"[TIAB] OR Glioblastoma*[TIAB] OR GBM[TIAB] OR Astrocytoma*[TIAB] OR "Glioblastoma"[Mesh] OR oligoastrocytoma*[TIAB] OR oligodendroglioma*[TIAB]) AND ("Diagnosis"[Mesh] OR "Biomarkers, Tumor"[Mesh] OR "Biomarkers"[Mesh] OR "diagnosis" [Subheading] OR diagnos* OR Identif* OR Prognos* OR marker* OR biomarker*)
Scopus	TITLE-ABS-KEY (microrna* OR mirna* OR mir OR micro-rna OR "Micro RNA" OR strna OR "Temporal RNA" OR pre-mirna OR pri-mirna OR microrna) AND TITLE-ABS-KEY ("primary central nervous system lymphoma" OR "CNS DLBCL" OR pcnsl OR "central nervous system lymphoma" OR "primary CNS lymphoma" OR glioblastoma* OR gbm OR astrocytoma* OR oligoastrocytoma* OR oligodendroglioma*) AND ALL (diagnos* OR identif* OR prognos* OR marker* OR biomarker*)
Web of Science	((TS= (((((((((microRNA*) OR (miRNA*)) OR (mir)) OR (micro-RNA)) OR ("Micro RNA")) OR (sirna)) OR ("Temporal RNA")) OR (pre-miRNA)) OR (pri-miRNA)) OR (microRNA))) AND (TS= (((((((((("primary central nervous system lymphoma") OR ("CNS DLBCL")) OR (PCNSL)) OR ("central nervous system lymphoma")) OR ("primary CNS lymphoma")) OR (glioblastoma*)) OR (GBM)) OR (astrocytoma*)) OR (oligoastrocytoma*)) OR (oligodendroglioma*)))) AND (ALL= (((((diagnos*) OR (identif*)) OR (prognos*)) OR (marker*)) OR (biomarker*)))
Embase	(microrna*:ti,ab,kw OR 'mirna*':ti,ab,kw OR 'mir':ti,ab,kw OR 'micro-rna':ti,ab,kw OR 'micro rna':ti,ab,kw OR 'strna':ti,ab,kw OR 'temporal rna':ti,ab,kw OR 'pre-mirna':ti,ab,kw OR 'pri-mirna':ti,ab,kw OR 'microrna':ti,ab,kw) AND ('primary central nervous system lymphoma':ti,ab,kw OR 'cns dlbcl':ti,ab,kw OR 'pcnsl':ti,ab,kw OR 'central nervous system lymphoma':ti,ab,kw OR 'primary cns lymphoma':ti,ab,kw OR 'glioblastoma*':ti,ab,kw OR 'gbm':ti,ab,kw OR 'astrocytoma*':ti,ab,kw OR 'oligoastrocytoma*':ti,ab,kw OR 'oligodendroglioma*':ti,ab,kw) AND (diagnos* OR identif* OR prognos* OR marker* OR biomarker*)
